# Use of multi-primer / multi-gene PCR method for the detection of *Mycobacterium tuberculosis* among female genital tuberculosis patients in India

**DOI:** 10.1186/1471-2334-14-S3-E12

**Published:** 2014-05-27

**Authors:** Venkanna Bhanothu, Jane Theophilus, Roya Rozati, AVikram Aiman

**Affiliations:** 1 Department of Zoology, UCS, Osmania University, Hyderabad-7, AP, India; 2 MHRT Hospital & Research Centre, Hyderabad, AP, India; 3 Gandhi Medical College, Hyderabad, AP, India

## Background

Female genital tuberculosis (FGTB) is a silent disease that evidences itself only when it is investigated. Demonstration of the etiologic agent by H & E staining or Z-N staining for acid fast bacilli, smear microscopy, and culture of sputum and other body fluid specimens were often less specific.

## Methods

A prospective case-control study was undertaken. A total of 302 endo-ovarian tissue biopsies collected from 202 infertile women highly suspected of having genital tuberculosis on laparoscopic examination and from 100 control women of reproductive age. All specimens tested by conventional/phenotypic methods were later compared with multi-gene/ multi-primer PCR method using four sets of primers for detection of *Mycobacterium tuberculosis* (MTB) in a single tube-single step reaction.

## Results

The conventional methods showed 99% to 100% specificity with a low sensitivity, ranging from 21.78% to 42.08% while H & E staining showed a sensitivity of 51.48%. Multi-gene PCR method was found to have a much higher sensitivity of 70.29% with MTB64 gene, 86.63% with 19kDa antigen gene at species and TRC4 element at regional MTB complex level and 88.12% with MPT59 α-antigen/32kDa protein gene at genus level. The specificity of multi gene/multi primer PCR was 100%.

## Conclusion

Multi-gene PCR was found to be a powerful technique for diagnosis and differentiation of mycobacterial infection among endo-ovarian tissue biopsies taken from infertile patients with FGTB. We suggest site specific sampling and amplification of the 19kDa antigen gene in combination with TRC4 element as a successful multi-gene/ multi-primer PCR method for the diagnosis of FGTB.

